# Increased Rate of Close-Kin Unions in the Central Andes in the Half Millennium Before European Contact

**DOI:** 10.1016/j.cub.2020.07.072

**Published:** 2020-09-07

**Authors:** Harald Ringbauer, Matthias Steinrücken, Lars Fehren-Schmitz, David Reich

**Affiliations:** 1 Department of Genetics, Harvard Medical School, Boston, MA 02115, USA; 2 Dept. of Human Evolutionary Biology, Harvard University, Cambridge, MA 02138, USA; 3 Department of Human Genetics, University of Chicago, Chicago, IL, USA; 4 Department of Ecology and Evolution, University of Chicago, Chicago, IL, USA; 5 UCSC Paleogenomics, University of California, Santa Cruz, Santa Cruz, CA 95064, USA; 6 UCSC Genomics Institute, University of California, Santa Cruz, Santa Cruz, CA 95064, USA; 7 Broad Institute of Harvard and MIT, Cambridge, MA, 02142, USA; 8 Howard Hughes Medical Institute, Harvard Medical School, Boston, MA 02115, USA.

## Abstract

Analyzing ancient DNA of the central Andes, Ringbauer et al. identify a markedly elevated rate of unions of closely related parents after ca. 1000 CE. This change of mating preferences sheds new light on a unique system of social organization based on ancestry (“ayllu”) whereby within-group unions were preferred to facilitate sharing of resources.

Spanish colonial sources describe how some groups in the central Andes practiced a unique system of social organization based on ancestry, whereby within-group unions were preferred to facilitate sharing of resources beyond the nuclear family. However, these sources do not quantify the prevalence or origin in time of this “ayllu” system. We provide new evidence by analyzing genome-wide data from 46 ancient Andean individuals for close kin unions. We detect a substantial increase in the rate of close-kin unions from 9% to 46% after ~1000 CE. This occurred after the decline of Wari and Tiwanaku cultures and at the start of an era of small-scale polities known as the Late Intermediate Period. Thus, the mating preferences instantiated in ayllu were widespread in Andean society and developed centuries before the expansions of the Inca state in the 15th century.

If a person harbors long stretches of DNA lacking variation between the two copies of the genome they inherited from their parents, so called “runs of homozygosity” (ROH), the only plausible explanation is that their parents are closely related, a signal that can be detected with genome-wide DNA. Applying a method that can use low coverage ancient DNA to make such measurements [1], we analyzed 46 ancient individuals from the Central Andean [2–4]. We detected the presence of long ROH at the level typical for offspring of 1st or 2nd cousins in 13 of 46 Central Andes individuals (Data S1A, Figure 1A). The rate increased from before 1000 CE where we observe it in 2 of 22 individuals (95% binomial confidence interval: 1.1–29.2%), to afterward when it occurred in 11 out of 24 (25.6–67.2%, p=0.0083; two-sided Fisher’s exact test). The rate is lower in present-day Andeans (Data S1B, Figure 1B, [S1-S7]): we detect long ROH in 2 of 86 (0.28–8.2%) Peruvians from Lima, and in 11 of 56 (10.2–32.4%) diverse other Andeans, with the latter signal largely driven by Aymara speakers from the Ventilla region of Bolivia [3] where we observe long ROH in 6 of 18 individuals. Without ancient DNA data from intermediate periods, however, we can not discern whether there was a continuously high rate of close kin unions in this region over the last 500 years.

We considered the possibility that the increased rate of close-kin unions in the five centuries before European contact could be an artifact of uneven sampling. However, the instances of consanguinity are widespread, occurring in 8 out of 11 Late Intermediate Period and Late Horizon sites (1–4 individuals each), and four large regions (Figure 1B). No close relatives were detected within the sample analyzed here [2], showing that the signal is not influenced by clusters of close kin. The signal is also not driven by urban elites: the individuals we analyzed were almost entirely rural (35 of the 37 individuals for which there is an archaeological assignment [2], and were largely commoners (as only three individuals from a single site are archaeologically assigned as elites; Data S1). Close-kin unions were known in the highest strata of Inca society, but our results could not be predicted by this as mating practices are often very different across social strata [5] and our signal dates to centuries before the Inca.

The onset of the period of increased close kin unions coincides with the decline of two major Middle Horizon societies (the Wari and Tiwanaku; ~700–1050 CE) that covered most of the Central Andes, and the beginning of the Late Intermediate Period (~1050–1440 CE) when there was a transition to smaller scale polities. It was only by the Late Horizon (~1440–1534 CE) that large-scale states arose again with the Inca who spread over large parts of western South America [6,7]. Our findings are notable in light of the “ayllu” social units described by the Spanish, whereby groups defined themselves at least in part through shared ancestry and preferred within-group marriages to keep resources within the community and to facilitate cooperation beyond the nuclear family (today, the word “ayllu” is used to describe some forms of social organization in the Andes, but it is unclear how similar these practices are to ancient ayllu) [5,8].

Archaeologists have documented an increased rate of collective burial practices including “Chullpa” funerary monuments during the Late Intermediate Period as evidence of new social systems becoming common in this period [5], and indeed an ancient DNA study has found evidence for an association of Chullpas to kinship networks by finding evidence for a patrilineally based family group in a Chullpa [9]. Our findings of an increasing rate of close kin unions across the Central Andes--a type of information that is impossible to glean from archaeological evidence alone--provides the first direct evidence for a qualitative change in the nature of kinship patterns in the prehistoric Central Andes, and dates it to the onset of the Late Intermediate Period. The fragmented socio-political units, reduced trade distances and intensified inter-group violence that distinguished the Late Intermediate Period from earlier times are all factors that could plausibly have favored a shift in social practices to maintain resources under local family control [6,7]. The Inca often incorporated pre-existing practices [10], which would be consistent with this practice persisting into the Late Horizon.

Future ancient DNA studies that include more localities of the Central Andes, as well as more time points and diverse burial contexts, would refine the understanding of the nature and causes of the shift in mate choice preferences we have documented here.

## Supplementary Material

Supplement

Data_S1 Data S1: Tabular information about the publicly available genetic data we analyzed in this study.
**Sheet A:** Compiled metadata for all 46 ancient individuals included in this study, results of ROH analysis, and manual assignment of rural versus urban status and elite versus non-elite status based on archeological context. **Sheet B:** Compiled metadata for all 142 present-day individuals included in this study, results of ROH analysis. **Sheet C:** Figure of inferred ROH blocks longer than 8 cM on the 22 human autosomes for each of the 46 ancient individuals we analyzed in this study (the 5 “IL” individuals are shotgun sequences, the rest 1240K enrichment data, see Sheet A). The depicted ages represent 95% radiocarbon date ranges if available and context date ranges otherwise. Chromosome length is depicted in genetic map units (Morgan).

## Figures and Tables

**Figure 1:**
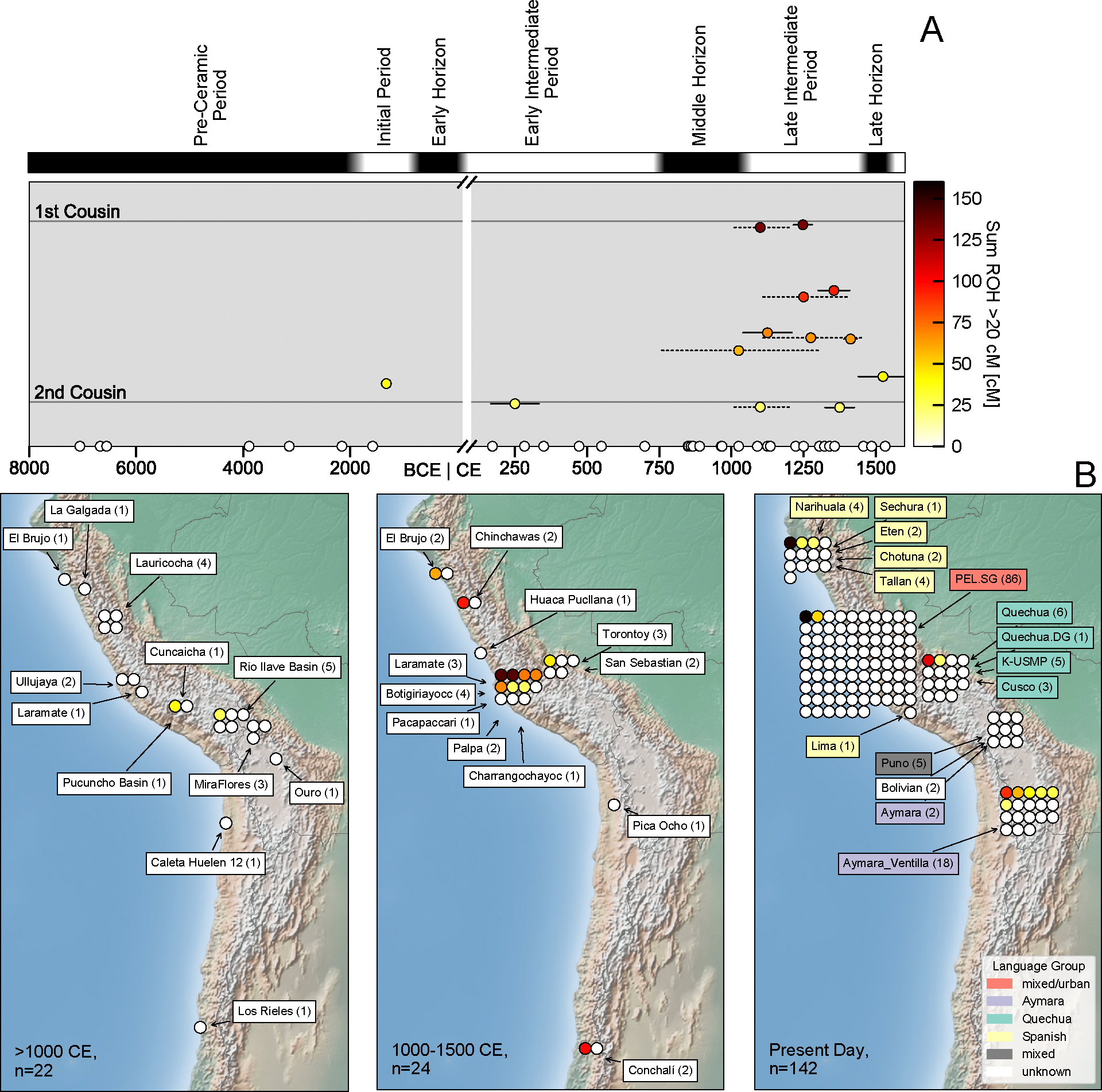
Long ROH in the Andean highlands. Ancient individuals with long ROH became more common after the onset of the Middle Horizon (ca. 1050 CE). **(A)** Each dot represents one ancient individual with sufficient data to make a measurement (at least 400,000 single nucleotide polymorphisms), and we show the sum of all ROH segments of at least 20 centimorgans, a threshold at which there is a high likelihood of the individual being an offspring of first or second cousins. We depict age uncertainties for the subset of individuals that have such long ROH (solid lines: 95% central intervals for radiocarbon dates, dashed lines: context date ranges). Age uncertainties for all individuals can be found in *Supp. Table 1*. **(B)** The geographic signal is widespread: we show individuals in North, Central and South Andean regions before (left) after ca. 1000 CE (middle). Right: Present-day individuals with at least partial indigenous ancestry, color-coded by language group.
