# Supplementary material for: Increased Rate of Close-Kin Unions in the Central Andes in the Half Millennium Before European Contact
Source: Curr Biol. Author manuscript; Available in PMC 2024 Nov 18. (PMC11571662; doi:10.1016/j.cub.2020.07.072)
Supplement: Supplement [file NIHMS2033507-supplement-Supplement.docx]

**Supplemental Information: Increased Rate of Close-Kin Unions in the Central Andes in the Half Millennium Before European Contact**

Harald Ringbauer, Matthias Steinrücken, Lars Fehren-Schmitz, David Reich


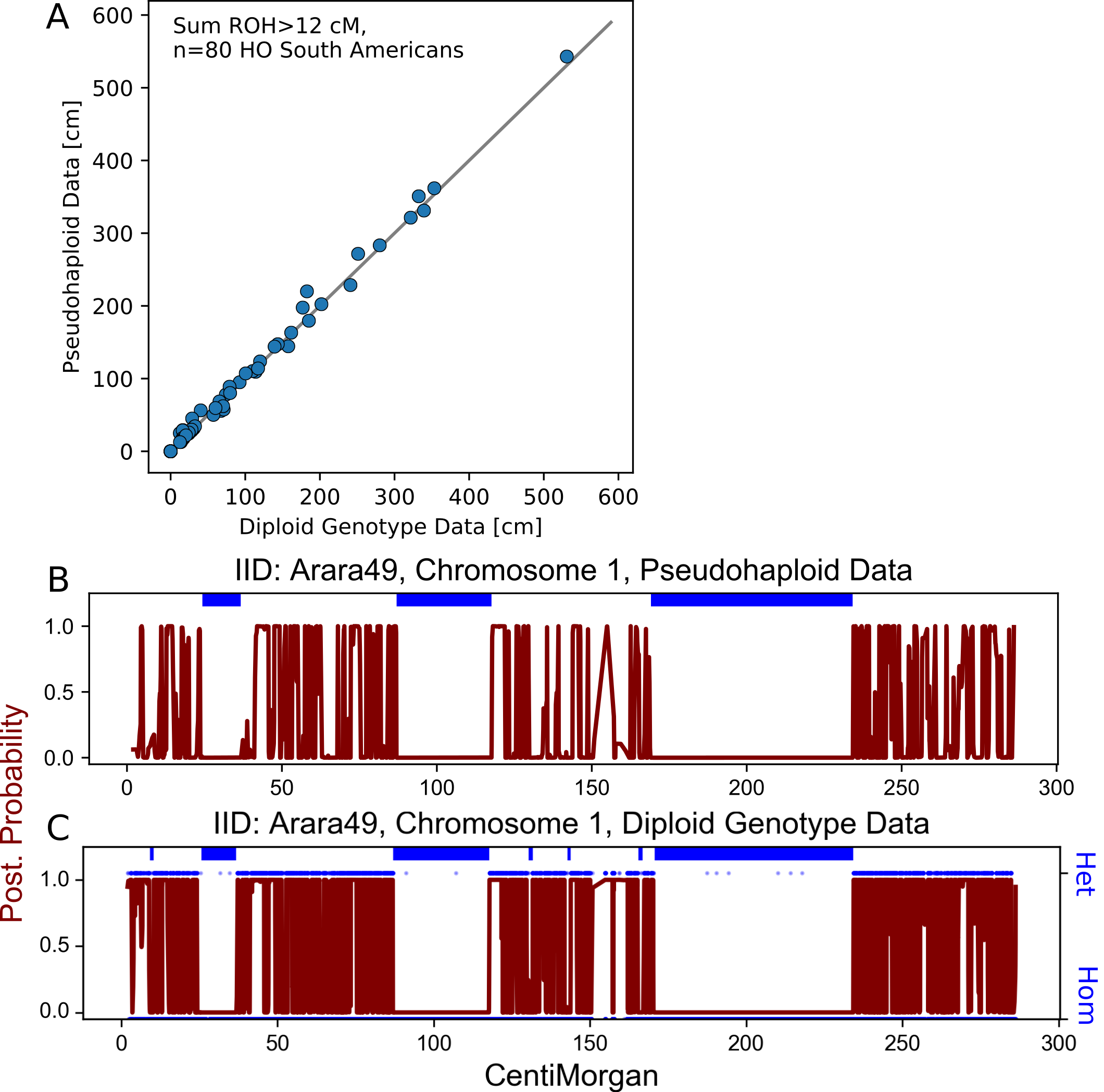
**Figure S1: Validation of the robustness of ROH calling on Andean data**. **(A)** We analyzed data from 80 present-day individuals from the Andes genotyped on the Affymetrix Human Origins SNP array. We infer ROH both on the full dataset, and on data downsampled to be similar in quality obtained for ancient DNA. We find good agreement for the statistic of the sum of all ROH >12 centimorgans (our actual analysis is even more conservative focusing on the 20 centimorgan threshold). **(B)** ROH calls (in blue) on data sampled to a single random allele on each SNP (typical for ancient DNA data) for chromosome 1 of one individual (Arara49). **(C)** ROH calls on diploid full genotype data for the same individual are qualitatively identical (we plot homozygote markers (down) and heterozygous markers (up), which visualizes long ROH).

**STAR Methods**

**RESOURCE AVAILABILITY**

***Lead Contact***

Further information and requests for resources should be directed to and will be fulfilled by the Lead Contact, David Reich (reich@genetics.med.harvard.edu).

***Materials Availability***

This study did not generate new unique reagents.

***Data and Code Availability***

All code used to produce the analysis and figures here is publicly available at *github.com/hringbauer/AndeanROH.* The Python package “hapROH” used to analyze the ancient DNA for ROH can be downloaded and installed via pip from *https://test.pypi.org/project/hapsburg/*

All ancient and modern DNA data are publicly available, and the meta-data describing the individuals is compiled in Data S1.

**METHOD DETAILS**

We applied the software *hapROH* (downloaded from <https://pypi.org/project/hapROH/>*,* version 0.1a4) to screen the ancient individuals for ROH. We used pseudo-haploid data on 1240K SNPs to analyze the eigenstrat files provided by the original publications. We used default parameters of hapROH, which are tuned to work well on 1240K SNPs. We report the total sum of ROH >4, >8, >12, >20 centiMorgan (Data S1), using the default genetic map of *hapROH,* and 5008 haplotypes from 1000 Genomes as reference.

We also manually annotated urban/rural and elite/regular for each individual (Data S1).

**QUANTIFICATION AND STATISTICAL ANALYSIS**

To compare the fraction of individuals that have long RO across two groups, we used Fisher's exact test to assess p-Values and report two-sided p-Values. We used the function *fisher_exact* of the python package *scipy.stats (*version 1.4.1.*).*

For 95% confidence intervals (CI), we report exact binomial confidence intervals, using an online tool available at <https://statpages.info/confint.html> *(*assessed May 10th 2020).

**Supplemental References**

S1. Barbieri, C., Barquera, R., Arias, L., Sandoval, J.R., Acosta, O., Zurita, C., Aguilar-Campos, A., Tito-Álvarez, A.M., Serrano-Osuna, R., Gray, R., et al. (2019). The current genomic landscape of western South America: Andes, Amazonia and Pacific Coast. Mol Biol Evol. Available at: https://academic.oup.com/mbe/advance-article/doi/10.1093/molbev/msz174/5539872 [Accessed August 11, 2019].

S2. Lazaridis, I., Patterson, N., Mittnik, A., Renaud, G., Mallick, S., Kirsanow, K., Sudmant, P.H., Schraiber, J.G., Castellano, S., Lipson, M., et al. (2014). Ancient human genomes suggest three ancestral populations for present-day Europeans. Nature 513, 409–413.

S3. Raghavan, M., Steinrucken, M., Harris, K., Schiffels, S., Rasmussen, S., DeGiorgio, M., Albrechtsen, A., Valdiosera, C., Avila-Arcos, M.C., Malaspinas, A.-S., et al. (2015). Genomic evidence for the Pleistocene and recent population history of Native Americans. Science 349. Available at: <http://www.sciencemag.org/content/349/6250/aab3884.abstract>.

S4. Mallick, S., Li, H., Lipson, M., Mathieson, I., Gymrek, M., Racimo, F., Zhao, M., Chennagiri, N., Nordenfelt, S., Tandon, A., et al. (2016). The Simons Genome Diversity Project: 300 genomes from 142 diverse populations. Nature 538, 201–206.

S5. Skoglund, P., Mallick, S., Bortolini, M.C., Chennagiri, N., Hünemeier, T., Petzl-Erler, M.L., Salzano, F.M., Patterson, N., and Reich, D. (2015). Genetic evidence for two founding populations of the Americas. Nature. Available at: http://www.nature.com/doifinder/10.1038/nature14895$\backslash$nhttp://www.ncbi.nlm.nih.gov/pubmed/26196601.

S6. Lindo, J., Haas, R., Hofman, C., Apata, M., Moraga, M., Verdugo, R.A., Watson, J.T., Llave, C.V., Witonsky, D., Beall, C., et al. (2018). The genetic prehistory of the Andean highlands 7000 years BP though European contact. Science Advances 4, eaau4921.

S7. 1000 Genomes Project Consortium. (2015). A global reference for human genetic variation. Nature, 526(7571), 68-74.
